# Immune status of the mother and early exposure through breastfeeding both influence the protection of the progeny from food allergy development to a major cow’s milk allergen

**DOI:** 10.1186/2045-7022-5-S3-P2

**Published:** 2015-03-30

**Authors:** Karine Adel-Patient, Sandrine Ah-Leung, Hervé Bernard, Valérie Verhasselt

**Affiliations:** 1 INRA, UR496, Unité d'Immuno-Allergie Alimentaire, IBiTecS/SPI/CEA de Saclay, Gif-sur-Yvette, France; 2 EA 6302 "Tolérance Immunitaire", Université de Nice Sophia-Antipolis, Hôpital de l'Archet, Nice, France

## 

Early tolerance induction is an attractive approach for primary prevention of food allergies. Using a mouse model, we aimed to assess the role of the immune status of the mother and the role of exposure during breastfeeding on the food allergy induced in the progeny.

Females BALB/c mice were either orally tolerized or orally sensitized to bovine β-lactoglobulin (BLG), a major cow’s milk allergen. Naïve mice were used as controls. Females were then mated with age matched naïve males. At delivery, pups were bred by their mothers or pups from sensitized/tolerised mother were replaced by pups from naive mothers to focus on the breastfeeding effect. Mothers were then exposed or not to BLG by gavage every other day until weaning. BLG, specific Ig and BLG-IgG immune complexes were assayed in breast milk. When 5-week old, progeny was experimentally sensitized to BLG and sensitization levels were assessed by measurement of BLG-specific IgE and IgG1. The allergic reaction was assessed by mMCP1 measurement in sera obtained 1hr after an oral challenge with BLG.

BLG-specific IgE, IgA and IgG1 and BLG-IgG1 immune complexes were detected only in milk from sensitized mothers. Exposition to BLG during breastfeeding increased these levels. Pups from and bred by naïve mothers exposed to BLG during breastfeeding demonstrated the same sensitization and allergic reaction levels than control pups. Conversely, a significant protection of the progeny was observed in pups from naïve or orally-sensitized mothers breastfed by orally sensitized mothers. The protective effect was enhanced if the mothers are exposed to BLG during breastfeeding, correlating with the level of IgG1-BLG complexes in breast milk. Protection of the progeny was less efficient when mother were orally tolerized and occurred only if there is exposure to the allergen during breastfeeding.

Altogether, these results thus demonstrate that protection of the progeny from food allergy depends on the immune status of the mother, i.e. naïve *vs* orally sensitized or tolerized. Importantly, exposition of the non-naïve mother to the allergen by the oral route during breastfeeding allows enhancing this protective effect.

